# Word position coding in reading is noisy

**DOI:** 10.3758/s13423-019-01574-0

**Published:** 2019-02-23

**Authors:** Joshua Snell, Jonathan Grainger

**Affiliations:** Aix-Marseille University & CNRS, Marseille, France

**Keywords:** Reading, Word recognition, Word position coding, Parallel processing

## Abstract

In the present article, we investigate a largely unstudied cognitive process: *word position coding*. The question of how readers perceive word order is not trivial: Recent research has suggested that readers associate activated word representations with plausible locations in a sentence-level representation. Rather than simply being dictated by the order in which words are recognized, word position coding may be influenced by bottom-up visual cues (e.g., word length information), as well as by top-down expectations. Here we assessed how flexible word position coding is. We let readers make grammaticality judgments about four-word sentences. The incorrect sentences were constructed by transposing two words in a correct sentence (e.g., “the man can run” became “the can man run”). The critical comparison was between two types of incorrect sentence: one with a transposition of the inner two words, and one with a transposition of the outer two words (“run man can the”). We reasoned that under limited (local) flexibility, it should be easier to classify the outer-transposed sentences as incorrect, because the words were farther away from their plausible locations in this condition. If words were recognized irrespective of location, on the other hand, there should be no difference between these two conditions. As it turned out, we observed longer response times and higher error rates for inner- than for outer-transposed sentences, indicating that local flexibility and top-down expectations can jointly lead the reader to confuse the locations of words, with a probability that increases as the distance between the plausible and actual locations of a word decreases. We conclude that word position coding is subject to a moderate amount of noise.

Very little prior research has explicitly investigated how humans encode the order of words during reading, and a lightning-speed summary of the history of reading research readily reveals why. First, a large chunk of reading research has focused on the recognition of single, isolated words (see, e.g., Grainger, 2008, for a review). Second, research on sentence and text reading has been dominated by the view that words are recognized in a serial, one-by-one fashion, so that knowledge of word order is simply implicated by the order in which recognized words are appended to sentence representations in memory. In short, the encoding of word order during reading has received close to no investigation because it was considered a given.

Currently, however, reading research is facing accumulating evidence that words are to some extent processed in parallel rather than serially. This necessitates, more than ever, the answering of a 10-year-old question: *How would a parallel processing system keep track of word order?* (Reichle, Liversedge, Pollatsek, & Rayner, 2009). In their opinion article, Reichle et al., the creators of the serial-processing E-Z Reader model, noted that a parallel-processing system is likely to recognize words out of order—for instance, when an upcoming word is much easier to recognize than the fixated word—and that it is unclear how the system would handle such occurrences. They then noted that “one possibility is that a buffer maintains word meanings, and that some mechanism re-orders out-of-order words”; however, a “problem with this solution is how such mistakes are detected without using comprehension difficulty to signal such occurrences” (pp. 116–117). Indeed, this “parallel-processing problem” (Snell, van Leipsig, Grainger, & Meeter, 2018) has long been a key argument in favor of serial processing―and perhaps rightfully so, if for no other reason than Occam’s razor. Surely the reading system could not be so complex as to engage in parallel processing?

## The reading system engages in parallel processing

The serial- versus parallel-processing debate has been guided by the assumption that if multiple words are processed in parallel, then information should be integrated across words, such that a word like, say, “dog” should be recognized faster if it is followed by “cat.” Such effects have been largely elusive in sentence reading (see Brothers, Hoversten, & Traxler, 2017, for a meta-analysis of the prior experimental literature), leading researchers to argue for serial processing. Recently, however, attention has been paid to the possibility that parallel processing might proceed without integrating semantic information across words. The key argument, as formulated by Snell, Meeter, and Grainger (2017), is that readers would have to be able to keep track of separate word identities, given that each word has a unique role in contributing to sentence comprehension. In this sense, the absence of so-called parafoveal-on-foveal effects would be logical even if parallel processing were true.

But how would one then directly evidence parallel processing? The solution offered by Snell et al. (2017) is that although parafoveal-on-foveal effects would, for aforementioned reasons, not be observable in direct measures of word recognition speed (e.g., fixation durations in sentence reading), parallel activated words might nonetheless jointly impact on measures invoked by certain tasks, such as when the reader is asked to make decisions or to report word identities. This idea has sparked the employment of paradigms beyond the realm of normal sentence reading, and has been further fueled by the conception that a successful theoretical framework of the reading system should be able to account for behavior in natural and artificial settings alike.

This endeavor has generated a set of results that can seemingly not be harmonized with a serial-processing framework, while on the contrary being quite logical under the assumption of parallel processing. First, syntactic and semantic categorization decisions to foveal target words are made faster when these words are flanked by syntactically or semantically congruent parafoveal words, as compared to incongruent words (Snell, Declerck, & Grainger, 2018; Snell et al., 2017). Crucially, these effects are established when the target and flankers are shown (simultaneously) for only 170 ms, which is shorter than the average time needed to recognize single words (Rayner, 1998). The fact that the syntactic and semantic characteristics of adjacent words nonetheless have an impact indicates that they must have been processed *during*, rather than *after*, target processing.

Second, using the novel rapid parallel visual presentation (RPVP) paradigm, Snell and Grainger (2017) found that when viewing four-word sentences for only 200 ms, readers were able to recognize any word with an accuracy of ~ 70% if the four words were syntactically coherent. A scrambled sequence of the same words, with the same target word having been presented at the same position, led to accurate recognition ~ 50% of the time (Snell & Grainger, 2017). This *sentence superiority effect* was perfectly equal across the four word positions, indicating that syntactic information was picked up from all words during the 200-ms presentation time, which in turn constrained the lexical identification of individual words.

How, then, would a parallel-processing system keep track of word order? Snell and colleagues (Snell, Declerck, & Grainger, 2018; Snell et al., 2017; Snell, van Leipsig, et al., 2018) have posited that a first glance at a sentence generates a spatiotopic sentence-level representation that comprises information about the number of to-be-recognized words in the perceptual span, as well as low-level visual information (e.g., word shapes and word lengths). Sublexical processing across multiple words would lead to the activation of multiple lexical representations, irrespective of their locations. These would subsequently be mapped onto plausible locations in the sentence-level representation, on the basis of bottom-up visual cues as well as top-down expectations (e.g., having recognized an article at Position 1, one might expect an adjective or noun at Position 2, and a word at Position 3 might constrain the lexical identification at Position 2 in a similar way).

**Fig. 1:**
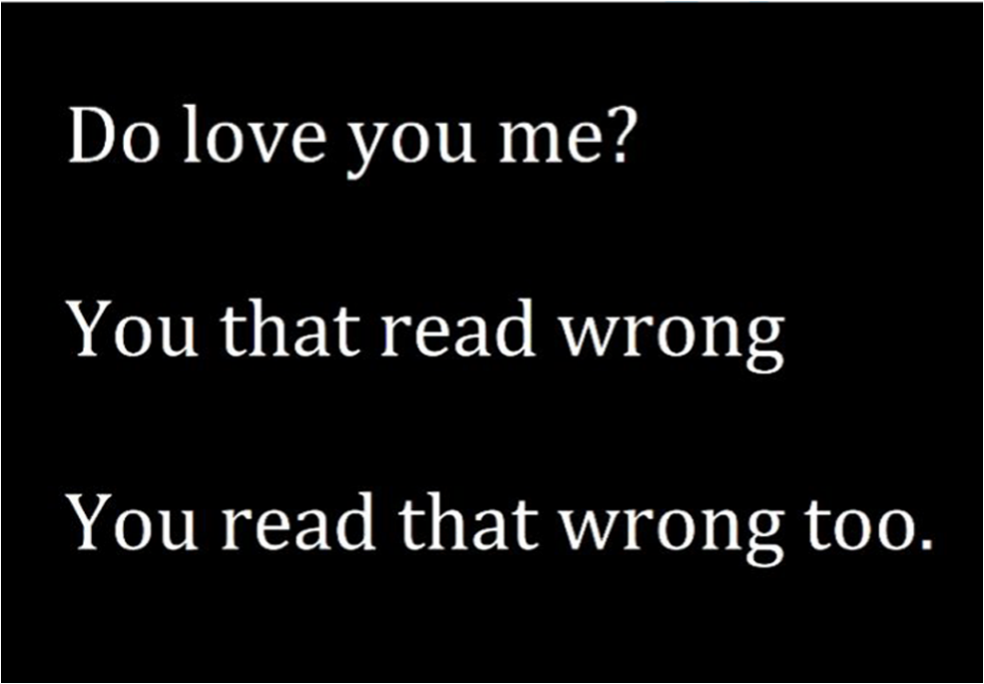
Illustration of the transposed-word effect

Recently we have investigated a phenomenon illustrated in Fig. 1. The figure shows that the reading system is in fact capable of doing what Reichle et al. (2009) deemed impossible: that is, to recognize words out of order―which, as they noted themselves, should not be possible under the assumption of serial processing. Testing the so-called *transposed-word* phenomenon in an experimental setting, Mirault, Snell, and Grainger (2018) let readers make speeded grammaticality judgments about sequences of words that could be either grammatically correct or ungrammatical. The crucial comparison was between two types of ungrammatical sequences: one that could be “corrected” by the system through the transposition of two words (e.g., “the ran dog slowly,” which could be transformed into “the dog ran slowly” by transposing “ran” and “dog”), and one that could not be corrected (e.g., “the was dog slowly”). Mirault et al. observed that readers had a much harder time classifying the former as incorrect, suggesting that the reading system retains some flexibility in the encoding of word position. Importantly, this transposed-word effect was observed in conditions in which the same words were used to create the two types of critical ungrammatical sequence, allowing us to control for effects driven by the properties of individual words and by local differences in grammaticality (see Table 1 in Mirault et al., 2018, for more details).

Following the logic of Reichle et al. (2009), the fact that readers can mix up the order of words may in itself prove to be problematic for the serial-processing assumption. It is important, nonetheless, to determine what exactly drives errors in word position coding. Figure 1 and the study of Mirault et al. (2018) seem to suggest that the reader’s top-down expectations largely dictate which word is associated with which position. This propels the assumption that words are recognized irrespective of location, and that word position coding proceeds largely through postlexical operations in working memory (Snell, Declerck, & Grainger, 2018; Snell et al., 2017; Snell, van Leipsig, et al., 2018).

At the same time, this does not exclude the possibility that the retrieval of location information is at least to some extent inherent to the word recognition process. Indeed, the only other previous study to our knowledge that has investigated word position coding showed that the transposition of two upcoming words in the parafovea induced a processing cost (Rayner, Angele, Schotter, & Bicknell, 2013). It may be that while readers sometimes err due to uncertainty or noise in the process of associating words with locations, this uncertainty is fairly limited. In technical terms, uncertainty in word position coding may be reflected by a relatively narrow Gaussian distribution. The latter scenario generates the following prediction: *The likelihood that readers err should increase as the distance between the plausible location and the actual location of a word decreases*. We tested this proposal in the present study.

## The present study

The present study builds on Mirault et al. (2018) in several important ways. First, although Mirault et al. tested the same words in two conditions across different sentences and different participants using a Latin-square design, different nonadjacent word combinations necessarily occurred in the two conditions, and these could have differed in terms of semantic coherence (e.g., “ran” and “slowly” might be experienced as more semantically coherent than “was” and “slowly,” thus biasing the reader to classify the sentence as grammatically correct). Second, it is not yet clear how flexible the process of word position coding truly is: Either words in the perceptual span may be recognized completely, irrespective of location (meaning that words are freely assigned to spatial locations), or alternatively, words may be to some extent inherently tied to spatial locations—for instance, through the reader’s knowledge about which letters belong to which spatial location (e.g., Grainger, Dufau, & Ziegler, 2016). As such, a word transposition error should be more likely to occur when the transposed words are adjacent than when they are nonadjacent.

We let readers make grammatical judgments about four-word sentences and, similar to the design reported by Mirault et al. (2018), compared performance between two types of ungrammatical sequence: one with a transposition of the inner two words (“the can man run”) and one with a transposition of the outer two words (“run man can the”). This manipulation allowed us to use the exact same set of words in the two types of ungrammatical sequence, while manipulating the distance between the transposed words. This is an important extension of the Mirault et al. study, in which distance was held constant (the transposed words were always adjacent) at the cost of having different word combinations in the two conditions.

We anticipated that, if location information is inherent to the recognition process, it should be harder to classify the inner-transposed sentences than the outer-transposed sentences as grammatically incorrect. Alternatively, if the reading system were organized such that activated words were assigned a spatial location completely freely (e.g., Snell, Declerck, & Grainger, 2018; Snell et al., 2017; Snell, van Leipsig, et al., 2018), there would be no difference between the inner- and outer-transposed sentences.^1^

Note that in our comparison of inner- versus outer-transposed sentences, we employed no true baseline condition. As such, if we were to observe no difference between the inner- and outer-transposed conditions, in the absence of all prior knowledge it would not be clear whether this were indeed due to complete flexibility, or the exact opposite—complete rigidity (with any word transposition yielding maximum cost, regardless of distance). The reason why we did not need a baseline to arbitrate between these two accounts is that the study of Mirault et al. (2018) has already evidenced flexibility in word position coding, rendering complete rigidity invalid as an explanation.

## Method

### Participants

A total of 24 participants from Aix-Marseille University gave informed consent to their participation in this study. All participants reported being nondyslexic, native speakers of the French language and having normal or corrected-to-normal vision. Participants received €5 as monetary compensation.

### Stimuli and design

From the 200 French four-word sentences used in the RPVP study of Snell and Grainger (2017), we used a subset of 120 stimuli in the present study. These stimuli had been tested on their semantic neutrality, as reflected by the words’ cloze probabilities deviating nonsignificantly from zero (Snell & Grainger, 2017). The word lengths ranged from three to five letters. For each stimulus, we made sure that both a transposition of the inner two words and a transposition of the outer two words rendered the stimulus ungrammatical.

All 120 stimuli were shown four times to all participants: once with an inner transposition, once with an outer transposition, and twice as the grammatically correct base sentence (to induce equal occurrence of the grammatical and ungrammatical sequences in the grammaticality judgment task). The 480 experimental stimuli were presented in random order.

### Apparatus and software

The experiment was implemented with OpenSesame (Mathôt, Schreij, & Theeuwes, 2012). Stimuli were presented on a gamma-calibrated 21-in. screen (1,024 × 768, 150 Hz), and responses were collected with an AZERTY-layout keyboard. Participants were seated at an 80-cm distance from the display, so that each character space subtended 0.35 deg of visual angle.

### Procedure

**Fig. 2:**
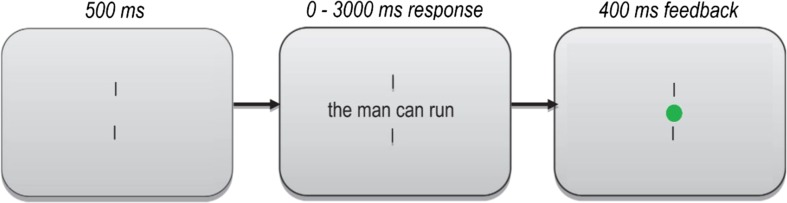
Trial procedure. The size of the stimuli relative to the screen is exaggerated in this example

Participants were seated in a comfortable office chair in a dimly lit room, where they received task instructions from the experimenter as well as visually onscreen. The trial procedure is shown in Fig. 2. Each trial started with a 500-ms fixation display comprising two centrally positioned vertical fixation bars on a luminance-neutral gray background. The fixation display was succeeded by a stimulus display that stayed on screen until the participant responded with a right- or a left-handed button press, for grammatical and ungrammatical sequences, respectively. Participants had a maximum of 3,000 ms to respond. Upon the participant’s response, a 400-ms feedback screen was shown, with a red or green dot to indicate incorrect or correct responses, respectively. Participants were offered a break on two occasions. The 480 experimental trials were preceded by eight practice trials for which we did not collect data. The experiment lasted approximately 25 min.

## Results

In the analyses of response times (RTs) as well as errors, we excluded trials with RTs beyond 2.5 *SD*s from the grand mean (2.66%). For the analysis of RTs, we also excluded incorrectly answered trials (9.97%).

The data were analyzed with linear mixed-effect models (LMMs) with items and participants as random effects. We isolated trials with inner- and outer-transposed sentences and added condition as a two-level fixed factor to the models. We used the maximal random-effect structure because the LMMs successfully converged when we included by-item and by-participant random slopes as well as random intercepts.^2^ We report *b* values, *SE*s, and *t* values (for RTs) or *z* values (for errors), with |*t*| and |*z*| > 1.96 being deemed significant (Baayen, 2008).

**Fig. 3:**
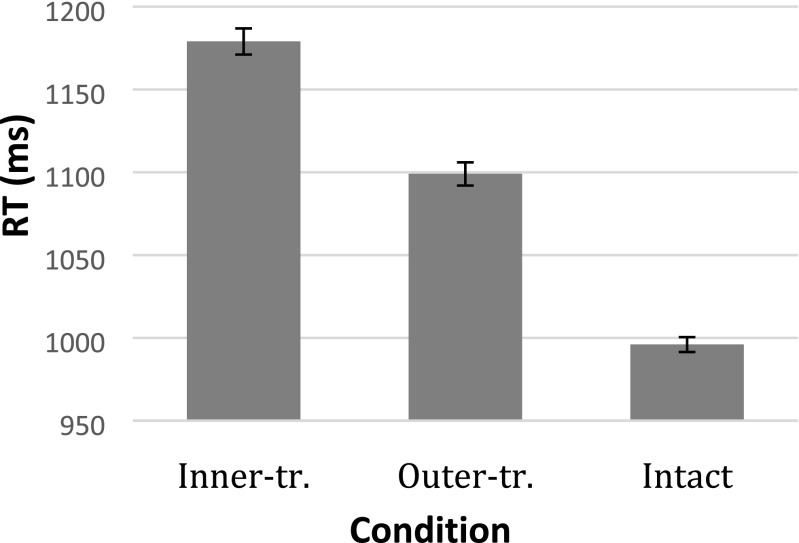
Average response times (RTs) per condition. Error bars indicate standard errors (*SE*s)

Average RTs are plotted in Fig. 3. A significant difference was observed between the inner- and outer-transposed sentences, such that readers were slower to classify inner-transposed sentences as grammatically incorrect: *b* = 90.36, *SE* = 17.77, *t* = 5.09.^3^ This effect was also expressed in the error rates, with more errors in the classification of inner-transposed sentences: *b* = 1.72, *SE* = 0.22, *z* = 7.75. Note that the intact sequences were included merely as part of the task design, and that contrasts between the transposed and intact sequences cannot be regarded as informative, since the fact that they belonged to different response categories constitutes a confound (see also note 1).

We established that the critical point of ungrammaticality was considerably earlier in the outer- than in the inner-transposed sentences. If readers processed words in a serial, left-to-right fashion, this would lead readers to respond more quickly to outer- than to inner-transposed sentences. To make sure that our effects were not driven by such a confound, we isolated all items wherein the critical point of ungrammaticality occurred at least at the second word (31.56% of trials).

When analyzing this subset of items (which had equal average points of ungrammaticality in both conditions: word 2.14 and word 2.15 for the inner- and outer-transposed sentences, respectively), our effects remained intact: for RTs, *b* = 95.00, *SE* = 20.69, *t* = 4.59; for errors, *b* = 1.40, *SE* = 0.33, *z* = 4.18. What is more, the critical point of ungrammaticality (which on each trial was set to 2, 3, or 4) was included in the LMM as a covariate and was not found to have a significant influence on either RTs (*b* = 16.16, *SE* = 16.87, *t* = 0.96) or error rates (*b* = – 0.01, *SE* = 0.19, *z* = – 0.07), hence suggesting that the words were not processed in a serial, left-to-right fashion. Additionally, the critical point of ungrammaticality was not found to modulate our condition factor: *b* = 32.81, *SE* = 34.08, *t* = 0.96.

In a final post-hoc analysis, we considered the possibility that participants could in some cases base their grammaticality judgments on the syntactic violation of a single transposed word in the outer-transposed sequences (e.g., on “the” in “run man can the”). Such cases occurred in outer-transposed sequences (in 34.2% of trials in the outer-transposed condition), but never in inner-transposed sequences, and this might have made decisions in the outer-transposed condition easier. Therefore, we reran our analyses a final time after excluding this subset of items. The pattern of effects remained intact: for RTs, *b* = 71.35, *SE* = 18.11, *t* = 3.94; for errors, *b* = 1.56, *SE* = 0.25, *z* = 6.25.

## Discussion

The present results are remarkably clear-cut: We found that readers had more difficulty classifying inner-transposed sentences as incorrect than outer-transposed sentences. Given that the stimuli comprised the same words across conditions and that the critical point of ungrammaticality was found to have no influence, we surmise that our effects were caused by differences in the distance between the words’ true locations, on the one hand, and their plausible locations, on the other. From these results, we infer that the retrieval of location information is to some extent inherent to the process of word recognition—the rationale being that if words were recognized completely irrespective of their location, there would have been no difference between the inner- and outer-transposed conditions.

It is important to stress that the conception that word position coding is noisy does not imply that the reader must consistently be ignorant or naïve about adjacent-word transpositions (in which case we would have established an effect in errors but not in RTs). Our account merely posits that a word is less likely to be associated with positions that are farther away from the word’s actual location, as is reflected in the Gaussian distribution by which uncertainty is usually modeled (e.g., noisy models of letter position coding: Gomez, Ratcliff, & Perea, 2008). As such, adjacent transpositions are met with more uncertainty than distant transpositions, hence making grammaticality judgments more difficult.

Because a confusion of word order was deemed to be impossible under the assumption of serial processing (Reichle et al., 2009), this study strengthens the conception that words are processed in parallel. Additional evidence against serial processing is provided by the fact that the critical point of ungrammaticality had no influence on decisions: Had readers processed words in a serial, left-to-right fashion (which, in this study, they indeed had ample time for), earlier points of ungrammaticality should have produced faster decisions.^4^

In light of the present study, it should be acknowledged that the process of word position coding is likely more intricate than was previously theorized by Snell and colleagues (Snell, Declerck, & Grainger, 2018; Snell et al., 2017; Snell, van Leipsig, et al., 2018). Specifically, lexical representations are not activated irrespective of location; instead, location information seems to be, to some extent, a component inherent to the recognition of individual words. A question that boasts immediate pertinence, then, is the extent to which this location information is dictated by bottom-up visual cues, on the one hand (e.g., readers might estimate which letters belong to which location in space; Grainger et al., 2016), or top-down expectations, on the other (e.g., upon starting to read a sentence, activated words from one syntactic category might be strongly favored for a given location over words from other syntactic categories; Snell, Declerck, & Grainger, 2018; Snell et al., 2017; Snell, van Leipsig, et al., 2018).

Having opened the investigation of word position coding, we welcome endeavors that will lead to the answering of such questions. For instance, to pinpoint the relative contributions of bottom-up and top-down processes, paradigms similar to the one reported here might manipulate the extent to which readers can rely on bottom-up versus top-down cues, respectively. One hypothesis would be that the effect of bottom-up cues should be attenuated as the number of letters shared by the two transposed words increases. Similarly, bottom-up cues should be weaker if the two transposed words are equal in length. Both of these manipulations would affect the discriminability of the two transposed words, with the rationale that a decrease in discriminability should lead to an increase in flexibility (and thus to more difficulty in classifying a transposed-word sentence as incorrect) if bottom-up visual cues play a key role.

Investigating the contribution of top-down influences might prove to be a bigger challenge and might necessitate the employment of different paradigms. One possibility would be to briefly present two consecutive sentences (note that the RPVP setup discussed in the introduction has already shown that readers can generate sentence-level representations within 200 ms of the sentence presentation time) and have readers make same–different matching decisions. The critical comparison would be between two conditions in which the second sentence was a transposed-word version of the first sentence: one condition in which the transposition of words led to a change in the sentence structure (e.g., “with these fine ladies”–“with fine these ladies”), and another in which the transposition did not lead to a change in sentence structure (e.g., “with pretty fine ladies”–“with fine pretty ladies”). If word position coding is influenced by top-down processes, then same–different matching decisions should be harder to make in the latter than in the former condition.

In anticipation of future research, we should also reflect on a potential shortcoming of the present study. It can be argued that visual acuity caused outer-transposed words (in particular the one that moved from Position 1 to Position 4) to have less of an influence than inner-transposed words. How this might have impacted on the present data is not clear. To us, it seems sensible that attenuated or slower processing of peripheral words should have led to more uncertainty, and thus poorer performance, in the outer-transposed condition. By this rationale, the better performance for the outer-transposed sequences reported here could not have been caused by confounds driven by visual acuity. In any case, this conception could be tested in a future experiment wherein a transposition of Words 1 and 3 is compared with a transposition of Words 2 and 4. Given that the transposed-word distances are equal between these two conditions, our account would predict no difference in task performance.

Finally, although we here claim to have provided a first investigation of word position coding, it is worth acknowledging the closely related research applying rational analysis to the study of language comprehension. Specifically, prior research has indicated that readers may maintain and act on uncertainty in the past linguistic input (Levy, Bicknell, Slattery, & Rayner, 2009). Bergen, Levy, and Gibson (2012) found that the ongoing generation of sentence-level representations can be subject to syntactic reanalyses (e.g., in anticipating or resolving garden-path constructions) that are incompatible with the language input. However, given that the sentences in this seminal study were presented one word at a time (in both the auditory and visual modalities), and that the syntactic uncertainty thus pertained to past rather than present linguistic information, the observed effects appear to have been driven by post-hoc operations altering the representations in working memory. In contrast, in the present study the sentences were displayed until the reader provided a response. In line with the transposed-word phenomenon illustrated in Fig. 1, this suggests that readers’ online interpretations of the relative positions of individual words are flexible.

In sum, in the present article we have shown that word position coding in reading retains some, but not complete, flexibility. We are confident that future research will help further elucidate the process of mapping activated words onto sentence-level representations.

## Footnotes

1 One might think that the latter scenario could also be tested by comparing intact to transposed sentences; however, this is not possible, since these two conditions are linked to different responses (analogously, one would not compare response times between words and nonwords in a lexical decision task). Hence, our investigation was solely based on comparing two transposed-word conditions that belonged to the same response category.

2 Because the question of whether simpler or more complex models should be preferred is still a matter of debate, we here note that all reported patterns persisted when using a model that included only by-item and by-participant random intercepts. A likelihood-ratio test showed that the complex model, in the case of the present results, does provide a better fit (*χ*^2^ = 82.52, *p <* .001).

3 An analysis of log-transformed RTs is sometimes preferred as log-transformed RTs tend to adhere better to the normal distribution assumption. We also analyzed log-transformed RTs and obtained virtually the same statistics. We therefore chose to report analyses of normal RTs here, because the *b* values can be more easily interpreted as such.

4 Proponents of serial processing might maintain that artificial reading tasks such as that in the present study cannot be used to make inferences about normal reading behavior. We would agree that different paradigms likely induce different types of behavior. However, we must also stress that our aim was not to describe reading behavior (which could be done solely through observing eye movements during text reading), but rather to infer the reading system’s core architecture. To suggest that processes of letter and word recognition, attention, and word position coding would be driven by an entirely different cognitive system in the present paradigm than in normal reading would raise more questions than it answers. Since serial- and parallel-processing frameworks have thus far done equally good jobs accounting for normal reading behavior, we maintain that the winning theory must be the one that can generate critical predictions both in- and outside the realm of text reading.
